# MicroRNAs in Autoimmunity and Hematological Malignancies

**DOI:** 10.3390/ijms19103139

**Published:** 2018-10-12

**Authors:** Mirco Di Marco, Alice Ramassone, Sara Pagotto, Eleni Anastasiadou, Angelo Veronese, Rosa Visone

**Affiliations:** 1 Ageing Research Center and Translational medicine-CeSI-MeT, 66100 Chieti, Italy; dmamirco@gmail.com (M.D.M.); alice.ramassone@unich.it (A.R.); sara.pagotto@unich.it (S.P.); a.veronese@unich.it (A.V.); 2 Department of Medical, Oral and Biotechnological Sciences (DSMOB), “G. d’Annunzio” University Chieti-Pescara, 66100 Chieti, Italy; 3 Harvard Medical School Initiative for RNA Medicine, Department of Pathology, Beth Israel Deaconess Medical Center, Harvard Medical School, Boston, MA 02215, USA; elenasta@gmail.com; 4 Department of Medicine and Aging Science (DMSI), “G. d’Annunzio” University Chieti-Pescara, 66100 Chieti, Italy

**Keywords:** autoimmunity, leukemia, lymphoma, microRNAs

## Abstract

Autoimmunity and hematological malignancies are often concomitant in patients. A causal bidirectional relationship exists between them. Loss of immunological tolerance with inappropriate activation of the immune system, likely due to environmental and genetic factors, can represent a breeding ground for the appearance of cancer cells and, on the other hand, blood cancers are characterized by imbalanced immune cell subsets that could support the development of the autoimmune clone. Considerable effort has been made for understanding the proteins that have a relevant role in both processes; however, literature advances demonstrate that microRNAs (miRNAs) surface as the epigenetic regulators of those proteins and control networks linked to both autoimmunity and hematological malignancies. Here we review the most up-to-date findings regarding the miRNA-based molecular mechanisms that underpin autoimmunity and hematological malignancies.

## 1. Introduction

The mechanisms that can drive to the loss of immunological tolerance involve both genetic and epigenetic factors. In a genetically susceptible host, environmental factors able to change cellular epigenetics could drive immunological disorders as autoimmune diseases (ADs). This paradigm could be fitting also for the hematological malignancies (HMs). However, tumorigenic process in HM comprehends the acquisition of specific oncogenetic alterations during cellular neoplastic transformation, whereas the genetic background predisposing to autoimmune diseases is usually congenital [1]. Why ADs and HMs are often associated seems to be imputable to epigenetic defects in the cells of the immune system [2], and growing evidence associates them explicitly to the epigenetic regulation of genes deputed to the individual immune homeostasis by microRNAs (miRNAs) [3]. The involvement of the miRNAs in ADs has been proven by the fact that enzymes deputed to their maturation process are dysregulated [4,5] and this is the first connection between ADs and HMs since the same enzymes are dysregulated in certain HMs as well [6,7,8]. Accordingly, several miRNAs are already found de-regulated in the most common ADs found in HMs such as, autoimmune systemic lupus erythematous (SLE), rheumatic arthritis (RA), systemic sclerosis (SSc) psoriasis (Ps), Sjögren’s syndrome (SS), idiopathic thrombocytopenic purpura (ITP) and autoimmune hemolytic anemia (AIHA) [9,10,11,12,13,14]. Among them, *miR-155* and *miR-21* stand out, which are two of the most frequently de-regulated miRNAs in HMs [15,16,17,18,19,20,21]. Therefore, although it is still challenging to understand which disease is more influential to the other one, these findings indicate new possible and targetable factors connecting ADs with HMs. Here we report the most recent discoveries on the miRNAs-based molecular mechanisms in those diseases that show clinical associations between the loss of immune tolerance and blood cancer [22,23]. 

## 2. MiRNAs in Autoimmune Diseases

Autoimmunity originates from the breakdown of self-tolerance. This leads to the imbalance between the activation of the lymphocytes and the mechanisms responsible for their control. The most common mechanisms associated with the autoimmunity include: (i) innate immune cell hyperactivation, such as dendritic and macrophage cells if functionally defective, can overstimulate T lymphocytes; (ii) lack of apoptosis of self-reactive B or T helper (Th) cells; (iii) curtailed presence of regulatory T cells; (iv) inflammation. MicroRNAs have been reported to have a role in each of these mechanisms.

### 2.1. Involvement of microRNAs in Innate Immune Cells Hyperactivation and Inflammation

Activate phenotype of the innate immune cells can over stimulate autoreactive T lymphocytes and produces pro-inflammatory cytokines. However, inflammation in autoimmune disease occurs also through the production of cytokines from other tissue-specific cells implicated in the pathology. Finally, chronic inflammation may result in fibrosis, which is common in certain ADs. Several miRNAs interfere with specific pathways related to the hyperactivation of the innate immune cells or pro-inflammatory cytokines and fibrotic tissue productions.

*MiR-155* acts into macrophages and dendritic cells from patients with ADs by altering their functions. It is overexpressed in macrophages resident in the membrane-lining layer and in CD14^+^ cells from synovial fluid of patients with rheumatoid arthritis (RA). Moreover, the enhanced expression of *miR-155* in RA monocytes reduces both the apoptosis by targeting caspase 10 (CASP10), apoptotic peptidase activating factor 1 (APAF1), and the expression of the chemokine C-C motif chemokine receptor 2 (CCR2), whereas increases the C-C motif chemokine receptor 7 (CCR7) and the secretion of C-C motif chemokine receptor 3/4/5 and 8 (CCL3, CCL4, CCL5, and CCL8). In CD14 from peripheral blood, it leads to the production of pro-inflammatory cytokines tumor necrosis factor and interleukin 6 (TNF, also known as TNFα and IL-6) as well as to the reduction of its direct target inositol polyphosphate-5-phosphatase D (INPP5D, also known as SHIP1), which is an inhibitor of inflammation. All these events favor the recruitment of leukocytes and the inflammation in RA [24,25,26]. SHIP1 is a target of *miR-155* also in dendritic cells (DCs). It was demonstrated that in a specific murine model the transfer of DCs pulsed with a self-antigen and matured following Toll-like-receptor (TLR) activation can induce autoimmunity. DCs over-expressing *miR-155* can break the immune tolerance also in the absence of TLR stimuli [27]. However, *miR-155* is part of the TLR signalling. Its expression increases by Toll-like receptor 7 (TLR7) stimuli in plasmacytoid DCs from New Zealand Black/White F1 hybrid (NZB/W F1) mice with symptomatic lupus, leading to the expression of the CD40 co-stimulatory molecule required to facilitate the T cell activation [28]. *MiR-155* can be considered as an effector of the inflammasome signaling in SSc. Its high expression reported in SSc lung fibroblasts is mediated by NLR family pyrin domain containing 3 (NLRP3) inflammasome and is required for the synthesis of collagen, whose accumulation induces fibrosis [29].

Other miRNAs such as *miR-574*, *let7b*, *miR-21*, *miR-130b*, *miR-302*, *miR-618*, and *miR-146a* were found to interfere with the type I IFN signaling. Plasmacytoid DCs from patients with SLE produce INFα upon TLR7 ligand stimulation through exosomes-delivered micRNAs (*miR-574*, *let7b*, and *miR-21*) [30]. *MiR-130b* and *miR-302d* are downregulated in kidney tissues from lupus nephritis or in monocytes from SLE and have as a target the interferon regulatory factor 1 and 9 respectively (IRF1; IRF9), which are involved in type I IFN response [31,32]. The expression of *miR-130b* is instead upregulated in SSc by transforming growth factor beta (TGF-β) signaling. In this context, it targets the peroxisome proliferator-activated receptor gamma (PPARγ), an antagonist of the pro-fibrotic TGF-β signaling and increases the level of fibrotic-related genes [33]. In SSc, *miR-618* promotes the secretion of IFNA1 in plasmocytoid DCs in SSc [34]. In monocytes from SLE patients, the type I IFN inhibits the maturation of *miR-146a* contributing to uncontrolled inflammation [35,36]. In mice, which have symptoms resembling those of the Sjögren’s syndrome, *miR-146a* was overexpressed in salivary gland cells and peripheral blood mononuclear cell (PBMC) altering the CD80 expression, which is a co-stimulatory molecule required for T cell activation [37]. However, the expression of *miR-146a* was associated with the risk of the SS and not of the SLE [38].

Altered expression of *miR-34a* and *miR*-*142-3p* in dendritic cells imbalances the T cell subsets. The activation of DCs is controlled by multiple mechanisms. *MiR-34a* plays a role mainly in CD1c positive DC cells from RA patients where it blows out the expression of the AXL receptor tyrosine kinase (AXL). This gene controls DCs activation through the induction of several genes among which the suppressor of cytokine signaling 1 (SOCS1). Indeed Kurowska-Stolarska et al. reported that the murine model *miR-34a* deficient is less susceptible to collagen-induced-arthritis and their DCs do not promote the T helper 17 cells (Th17) response [39]. Similarly, *miR-142-3p* furthers the pro-inflammatory function of monocyte-derived-DCs (moDCs) of patients with SLE and makes them more able to attract CD4^+^ T cells. Moreover, co-cultures of moDCs carrying *miR-142-3p* with allogenic T cells have reduced regulatory T cells (Treg), IL-10 and increased IL-17, suggesting that this miRNA fosters the T cell imbalance in this disease [40].

*Let-7c* contributes to the activated phenotype of DCs derived from *Blimp1^−/−^* mice, which is a key repressor of B and T lymphocytes. These mice develop pathological characteristics similar to that of SLE in patients. Kim, S.J. et al. [41], report that PR domain containing 1, with ZNF domain (PRDM1, also known as BLIMP1) suppresses *let-7c*, which in turn targets *Blimp1.* They also demonstrated that this miRNA targets the *SOCS1* gene and increases the secretion of pro-inflammatory interleukins establishing a connection between BLIMP1 and inflammation in DCs [41]. In a different murine model of SLE, *miR-150* abrogates the activated phenotype of DCs by interfering with the triggering receptor expressed on myeloid cells 1 (TREM1) signaling, which induces cytokines production after TLR-4 stimulation [42]. On the contrary, in biopsies from lupus nephritis patients, *miR-150* is overexpressed. In this context, it has a role in the fibrosis that characterizes this disease in that *miR-150* targets SOCS1, which also has an anti-fibrotic role in proximal tubular and mesangial cells [43]. In systemic sclerosis, *miR-150* could assume an antifibrotic role. Heindryckx et al. reported an indirect suppressive effect of *miR-150* on αSMA in human fetal lung fibroblasts with consequent inhibition of their differentiation in myofibroblasts. *MiR-150* maturation is regulated by the endoplasmic reticulum to nucleus signaling 1 (ERN1, also known as IRE1α) ribonuclease domain; however, another study proposed the DNA methylation as the mechanism that downregulates this miRNA in SSc [44,45].

*MiR-483-5p*, *miR-202-3p*, *miR-30b*, *miR-21*, *miR-7*, *miR-196a* and *miR-29a* seem to be part of the fibrotic process that characterizes the systemic sclerosis by modulating fibrosis-related proteins. Among these, *miR-29a* reduces the level of the BCL2 and B-cell lymphoma-extra-large (BCL-xL) antiapoptotic proteins; TGF-beta activated kinase 1 (MAP3K7) binding protein 1 (TAB1), an effector of the TGF-β pathway, TIMP metallopeptidase inhibitor 1 (TIMP1) and TNFα. So, *miR-29a* expression results in death of activated fibrobalsts from SSc together with increase of the antifibrotic matrix metallopeptidase 1 (MMP1) protein. *MiR-196a* is negatively modulated by TGF-β signaling in SSc fibroblasts and its overexpression reduced type I collagen. Another study has reported similar results [46,47,48,49,50,51,52,53,54].

MiRNAs, such as *miR-31*, *miR-10a-5p*, and *miR-17* assume a functional role in autoimmune disease by interfering with the nuclear factor NF-κB pathway. The high expression of *miR-31* found in psoriasis keratinocytes is due directly to the pro-inflammatory cytokines-mediated activation of the NF-κB pathway and leads to the reduction of the protein phosphatase 6, a negative regulator of the cell cycle progression. *MiR-31* targets also a negative regulator of the NF-κB pathway, the serin/threonine kinase 40 (STK40) affecting the production of chemokines and cytokines in human primary keratinocytes [55,56]. Similarly, *miR-10a-5p* is involved in a circuitry that at the end modulates the NF-κB-mediated production of inflammatory cytokines in RA. Indeed, TNFα and IL-1β decrease its expression in synovial fibroblast from RA patients probably through the NF-κB regulation of the YY1 transcription factor (YY1) repressor. In turn it targets several genes associated with the NF-κB pathway, such as interleukin 1 receptor-associated kinase 4 (IRAK4), mitogen-activated protein kinase kinase kinase 7 (MAP3K7, also known as TAK1) and beta-transducin repeat containing E3 ubiquitin protein ligase (BTRC). This mechanism is most probably responsible for the reduced secretion of pro-inflammatory cytokines (IL-6 and IL-8, TNFα and IL-1β) by fibroblast-like synovial cells after *miR-10a-5p* enhanced expression [57,58]. In the same cellular system, *miR-17* through stimulation by TNFα, promotes poly-ubiquitination of TNF receptor associated factor 2 (TRAF2), baculoviral IAP repeat containing 2 and 3 (BIRC2/BIRC3, also known as cIAP1 and cIAP2 respectively) and their degradation. This impairs the TNFα mediated nuclear translocation of the p65 subunit, part of the NF-κB complex, the Jun proto-oncogene, AP-1 transcription factor subunit (c-JUN) and signal transducer and activator of transcription 3 (STAT3) activity and the consequent production of IL-6, IL-8, matrix metallopeptidase 13 and 1 (MMP-13; MMP-1) [59]. In psoriasis *miR-17* and the other members of the *miR-17-92* cluster induce the release of C-X-C motif chemokine ligand 9 and 10 (CXCL9; CXCL10) from keratinocytes by targeting SOCS1, thus increasing inflammation because of the T cells attraction [60]. Moreover, as a result of *miR-146b* and *miR-10b*, keratinocytes in psoriasis produce less level of the atypical chemokine receptor 2 (ACKR2), a scavenger of pro-inflammatory chemokines [61].

Finally several miRNAs (*miR-486-3p*, *miR-155*, *miR-138*, *miR-181b*, *miR-125b*), found deregulated in psoriatic lesions, are involved in lack of apoptosis, abnormal differentiation and hyperproliferation of keratinocytes that secrete factors which contribute to the chronic inflammation [62,63,64,65,66]. 

### 2.2. Involvement of MicroRNAs in Autoreactive B Cells

B lymphocytes play a pivotal role in many ADs via their production of self-antibodies that may forms immune complexes responsible for organ-specific or systemic ADs. Prominent is also their function as antigen-presenting-cell (APC) through which they present the self-antigens to T lymphocytes inducing the expansion of an autoreactive T clone. Several miRNAs have been reported to influences these processes. 

*MiR-155* is elevated in B lymphocytes of SLE patients. Its role was investigated in Fas cell surface death receptor (FAS) deficient mice (Fas^lpr^), which are prone to develop a lupus-like disease. The authors demonstrated that lack of *miR-155* in this model, arrests the persistent hyperactivation of autoreactive B cells and the production of IgG autoantibodies, resulting in an ameliorated renal pathology. Mechanistically co-aggregation of the inhibitory receptor Fc gamma RIIB and B-cell receptor (BCR) in absence of *miR-155* resulted in an increase of its target SHIP1, whose lack stimulate the production of IgG antinuclear autoantibodies [67]. Physiologically, *miR-155* is important in B cells also for the extrafollicular and germinal center response, and its depletion impairs the capacity of B cells to produce high affinity IgG1 antibodies, by targeting Spi-1 proto-oncogene (SPI1, also known as PU.1). Indeed, PU.1 overexpression reduces IgG cell producing [68]. This *miR-155*-mediated mechanism is relevant also in B cells from synovial fluid of patients with RA after they were stimulated with CD40L, TNF superfamily member 13b (TNFSF13B, also known as BAFF), IL-21, and anti-IgM, suggesting that in this system depletion of *miR-155* alone is not sufficient to reduce IgG antibodies [69]. *MiR-155*-depleted murine models further confirm the link between this miRNA and the IgG production in that after exposure to collagen the amount of anti-collagen specific antibodies was reduced as compared to the control [26]. In the context of autoimmunity, a genetic variant in the *BAFF* gene with a shorter 3’UTR was associated with SLE. This genomic aberration resulted in an increased production of soluble BAFF, a potent regulator of humoral immunity, as ensuing of a miRNA inhibition escape [70]. In the same pathological context, a regulatory circuitry involving *miR-1246* controls over-activation of B cells. Loss of *miR-1246* results in an increase of its target implicated in B cell development, early B cell factor 1 (EBF1), B cells hyperactivity, co-stimulatory surface molecules such as, CD40, CD80, and CD86. Moreover the EBF1 expression leads to abnormal activation of AKT signaling and consequent repression of tumor protein p53 (TP53) activity including down-regulation of *miR-1246* [71]. Other miRNAs regulate the expression of co-stimulatory molecules as *miR-146b-5p* that increases the expression of CD80 potentiating B-T cells synapse in patients with CLL associated with autoimmune hemolytic anemia (AIHA) [14]. 

In contrast, low levels of *miR-142-3p* and *miR-142-5p* in SLE CD4^+^ T cells lead to an increase of IL-10, SH2 domain containing 1A (SH2D1A) and CD84 protein levels, promoting B cells hyper responsiveness in SLE [72]. *MiR-30a* was found elevated in SLE patients, playing a pivotal role in B cells proliferation and production of IgG antibodies. Its effect is exerted by direct repression of Src family tyrosine kinase (LYN) proto-oncogene, a mediator of the BCR complex. Intriguingly the expression of LYN is reduced in SLE patients [73]. By suppressing several targets involved in BCR signaling, such as growth arrest and DNA damage inducible alpha (GADD45A), phosphatase and tensin homolog (PTEN) and BCL2 like 11 (BCL2L11), upregulation of *miR-148a* promotes survival of immature B cells in a lupus mouse model [74]. In the same disease, *miR-326* is over expressed, playing a critical role in hyperactivity of B cell and autoantibodies secretion by repressing ETS proto-oncogene 1, transcription factor (ETS1), a negative regulator of B cells differentiation [75]. Another miRNA, *miR-146a* downregulates FAS expression in germinal center B cells leading to an autoimmune disorder in transgenic mice that resembles the lymphoproliferative syndrome (ALPS) in human [76]. Enhanced *miR-21* in normal T cells affects programmed cell death 4 (PDCD4), an inhibitor of genes involved in immune response that is very low expressed and negatively correlates with *miR-21* in T cells from SLE patients. In these cells depletion of *miR-21* reduces their proliferation and CD40L expression, affecting their capacity to drive the differentiation of B cells with consequently limited IgGs production [77]. Similarly, in a chronic graft versus host disease (GVHD) model of SLE generated by transfer of splenocytes from 6.C-H2bm12/KhEg mice into BL6 or BL6 *miR-21*^−/−^, lack of *miR-21* showed a drastic reduction in autoantibody titers, CD40:CD40L and CD28:CD80/86 stimulation signals [78]. In a different model of chronic GVHD, in which mice develop symptoms similar to that of patients with scleroderma, the absence of *miR-17-92* cluster in both B and T lymphocytes ameliorated the disease. *miR-17-92* in B cells was required for B cell autoantibodies production, while in T cell positively influenced the Th17 differentiation [79]. *MiR-150* shows an inverse correlation with the expression of MYB proto-oncogene, a transcriptional regulatory factor whose activation was found high in B-lymphocytes of AIHA/Evans syndrome [80].

### 2.3. Role of MicroRNAs in Effector and Regulatory T Cell Imbalance

Imbalance of the T lymphocytes subsets is a common pathogenic feature in autoimmune diseases. This occurs as a consequence of either expansion of a specific T helper clone subtype or dysregulation of mechanisms that lead to the differentiation of regulatory T cells. Indeed, Tregs are deputed to both halting the immune response and maintaining self-tolerance by monitoring self-reactive Th cell expansion. *MiR-155* stands out as one of the miRNAs mostly involved in T cell type imbalance in ADs. Physiologically *miR-155* participates in the development of thymus Treg (tTreg) and peripheral Treg through positive feedback with the C-type lectin receptor CD69. Depletion of CD69 in Treg cells abolished signal transducer and activator of transcription 5 (STAT5A, also known as STAT5) activation and *miR-155* transcription. In turn, the decrease of *miR-155* also reduces STAT5 activity and its binding to the forkhead box P3 (FOXP3) gene promoter, as well as CD69, thus impairing Treg cells development [81]. The role of *miR-155* in this context seems to be controversial since a study demonstrates the upregulation of this miRNA in Treg cells with aberrant phenotype of mice with a lupus-prone disease. *MiR-155* reduced the level of the L-selectin CD62L, which is required for a more potent suppressive effect of Treg [82]. In stimulated PBMCs from juvenile SLE patients, a different cellular context but similar disease, *miR-155* is downregulated. Restoring its expression results in a block of protein phosphatase 2 catalytic subunit alpha (PPP2CA), which is a critical immune modulator and a negative regulator of interleukin-2 (IL-2) [83]. Similarly, *miR-31* positively influences the IL-2 pathway by affecting nuclear factor of activated T cells 1 (NFATC1) activity [84]. Further, *miR-155* influences the pro-inflammatory phenotype of T cell through its aberrant expression in Treg and T helper cells in RA. Enforced expression of *miR-155* in Treg increases their production of IL-17, interferon gamma (IFNG) and TNF [85]. Indeed, mice lacking *miR-155* did not develop collagen-induced arthritis and had impaired Th17 cells activation and polarization through lesser levels of IL-22 and IL-17 [18].

*MiR-21* also causes T cells subsets imbalance. In a *miR-21^−/−^* murine model lupus-prone in which a chronic GVHD was induced, an expanded Treg compartment with concomitant reduction of co-stimolatory molecules and consequent decrease of Th17 cells were found [78]. Moreover the silencing of *miR-21* enhances the apoptosis rate of activated T helper cells in psoriasis context [86]. *miR-21* was found overexpressed in a murine model of autoimmune cholangitis generated by altering the TGF-β pathway and contributed to the production of the pro-inflammatory cytokines from T cell subsets. However, in PBMC from RA patients *miR-21* is decreased and its expression was inversely correlated with that of its target STAT3 that promotes Th17 differentiation and directly correlated with STAT5 activation that instead promotes Treg polarization [87]. *MiR-21* as well as *miR-148a*, *miR-126* and *miR-29b* interfere with the methylation of autoimmune-related-genes. *MiR-21* affects indirectly DNA methyltransferase 1 (DNMT1) expression in a lupus-prone murine model, by targeting RAS guanyl releasing protein 1 (RASGRP1*)*, linked to autoimmune disease. This elicits demethylation of CD70 and integrin subunit beta 2 (ITGB2, also known as LFA-1) promoters, thus increasing their gene expression. Similar effects were induced by *miR-148a*, *miR-126* and *miR-29b* that negatively modulate DNMT1 expression, thereby causing B and T cells overstimulation [88,89,90].

In psoriasis, *miR-210* promotes the development of pathogenic Th17 and Th1 but inhibits Th2 by repressing STAT6 and LYN. It contributes to the disease also decreasing FOXP3 in Treg, thereby compromising T cell subsets [91,92]. *MiR-146a* expression is down-regulated in PBMCs from patients with ITP and inversely correlated with Th17 cells. Overexpression of this miR contributed to Treg polarization. In accord with this, deficiency of *miR-146a* breaks the immunological tolerance and induces a strong IFNG response that elicit immune lesions in several organs of mice [93,94].

An aberrant expression of *miR-125a-5p* has been implicated in ITP. A long non-coding RNA, MEG-3, inhibits *miR-125a-5p*. Treatment with dexamethasone, commonly used as ITP therapy restored the expression of *miR-125a-5p* in ITP CD4^+^ T cells, likely by suppressing MEG-3; it also promoted Treg differentiation. The same authors showed, that the expression of *miR-125a-5p* repressed CXCL13 and its function in immunological response activity [95,96]. The cluster *miR-183/96/182* influences the *FOXP3* transcription by negatively regulating the transcription factor forkhead box O1 (FOXO1) and promoting Th17 cell polarization [97]. In Treg cells from SLE patients, *miR-326* inversely correlated with ETS1*,* a negative regulator of Th17 [98].

Another important player is *miR-23b*. This miRNA is part of a circuitry in which it is suppressed by IL-17 and reciprocally inhibits IL-17, together with TNFα, and pro-inflammatory cytokines. This occurs because of the *miR-23b* targeting of TAB2, TAB3, and conserved helix-loop-helix ubiquitous kinase (CHUK, also known as IKK-α) [99]. *MiR-138* was reported to be downregulated in the context of CD4^+^ T cells from psoriasis patients. Forced expression of this miRNA determined the downregulation of runt related transcription factor 3 (RUNX3*)*, restoring the Th1/ Th2 balance [100].

## 3. MiRNAs in Hematological Malignancies

Hematological malignancy is a collective term for grouping those neoplastic conditions of the hematopoietic and lymphoid tissues, which result in leukemias, lymphomas and myelomas. We will focus on leukemias and lymphomas since have been found concomitant with autoimmune diseases. The crucial role of miRNAs in the maintenance of the physiologic functions and diseases of blood and lymph system is well established nowadays [3,17,101,102,103].

Moreover, increasing evidence describe the central role of miRNAs in shaping the tumor microenvironment (TME) [104,105]. For instance, leukemia-derived exosomes transfer miRNAs to the surrounding microenvironments leading to enhanced angiogenesis, transformed stromal cells and immune suppression, which in turn favors the leukemic cells proliferation by suppression the normal hematopoiesis [106,107,108].

### 3.1. Chronic Lymphocytic Leukemia (CLL)

The most frequent deleted chromosome region in CLL, the 13q14, contains the miRNA cluster *miR-15a*/*miR-16-1* [109]. The down-regulation of *miR-15a* and *miR-16-1*, as a consequence of 13q14 deletion, contributes to CLL development [110,111,112] by deregulating essential pro- and anti-apoptotic factors as BCL2 and TP53 [110,113]. Their restoration in CLL cells affects the cell growth and tumor regression in a mouse model [114]. Recently, we identified an allele-specific transcriptional regulation of *miR-15a* and *miR-16-1*, which involves both the RNA polymerase II (RPII) and the RNA polymerase III (RPIII), recruited on a cryptic promoter upstream the miRNAs. In patients carrying the 13q14 deletion, the RPIII-mediated transcription was unmasked when the deletion occurred on the RPII-transcribed allele. Moreover, in our subset of patients, the RPIII-mediated transcription of *miR-15a* and *miR-16-1* was associated with a poor prognostic marker of CLL [115]. The 13q14 deletion is not the only cause of the *miR-15a* and *miR-16-1* down-regulation: for instance, a point mutation downstream the *miR-16-1* in the New Zealand Black (NZB) CLL mouse models affects the processing of *primiR-15a/16-1* to *pre-miR-15a/16-1*, thus leading to decreased expression of those miRNAs [116]. A common pathogenic pathway that link *miR-15a/16-1* cluster, *miR-34b/c* cluster and tumor protein p53 with 13q, 11q, and 17p deletion has been reported in CLL. Enforced expression *of miR-15a/16-1* reduces Tp53 and BCL-2 and increases of miR-34 family, which in turn target Zeta chain of T cell receptor associated protein kinase 70 (ZAP-70). Moreover, Tp53 can induce the expression of these miRNAs [117].

*MiR-150* is the most abundant miRNA in CLL, and its lower expression in CLL patients is associated with better prognosis of the disease. Accordingly, *miR-150* influences the BCR signaling by targeting GRB2 associated binding protein 1 (GAB1) and forkhead box P1 (FOXP1) [118]. 

*MiR-181b* is downregulated during the progression of CLL [119]; its enforced expression slows tumor expansion and prolongs the survival of Eμ-TCL1 transgenic mice [120]. Otherwise, *miR-21* is up-regulated in CLL [121] and associated with poor prognosis [20] and drug-resistance [122]. ZAP-70, whose high levels predict poor prognosis in CLL [123], affects the transcription of *miR-21*. Its overexpression increases the levels of *miR-21* via mitogen-activated protein kinase 1 (MAPK1, also known as ERK) and STAT3 signaling. Consequently, the noted targets of *miR-21* like the tumor suppressors phosphatase and tensin homolog (PTEN), PDCD4 and protein inhibitor of activated STAT 3 (PIAS3) are down-regulated, impairing cell survival [124]. *MiR-21* is also up-regulated by the IL-4 [125]. Several studies reported that the *miR-155* is over-expressed in CLL [126,127,128] and its overexpression correlates with un-mutated immunoglobulin heavy variable (IGHV) genes status and high levels of ZAP-70 [126]; the involvement of *miR-155* in promoting a B-cell disease was also underlined in mice models; indeed Eµ-miR-155 transgenic mice developed a high-grade B-cell malignancy [129]. High levels of *miR-155* were found in lymph node proliferation centers, in which the neoplastic cells undergo to activation and proliferation [130]. Moreover, *miR-155* enhances the BCR signaling [131], which is also involved in the development of CLL [132]. Furthermore, *miR-155* influences the chromosomal instability by direct down-regulation of centromere protein F (CENPF), zw10 kinetochore protein (ZW10) and BUB1 mitotic checkpoint serine/threonine kinase (BUB1) in CLL cell line [133].

### 3.2. Acute Myeloid Leukemia (AML)

High expression of *miR-155* was found in AML patients harboring the fms related tyrosine kinase 3 (FLT3) internal tandem duplication (ITD) (FLT3-ITD) [134,135,136], a poor prognostic marker of the disease [137]. *MiR-155* is up-regulated by the FLT3-ITD signaling pathway and affects cell proliferation. Silencing of *miR-155* induces apoptosis in the FLT3-ITD-associated cells by increasing the myeloid transcription factor PU.1 [138]. *MiR-155* promotes cell proliferation also by affecting the interferon (IFN) response. Its inhibition in FLT3-ITD^+^ AML cell lines or primary samples increases the IFN response and decreases cell proliferation [139]. Of note is the dose-dependent effect of *miR-155* in AML. Initially, the high expression of the miRNA reduces proliferation of AML cells whereas, after its long-term transduction, a selection of cells showing an intermediate expression of *miR-155* displays increased tumor burden in mice [140]. Another intriguing mechanism of *miR-155* in AML is driven by exosomes released by AML cells, which transport the miRNA to hematopoietic stem cells and progenitor cells; the release of *miR-155* in these cells suppresses the hematopoietic functions by targeting the transcriptional activator MYB [141].

In AML, the functional relevance of the *miR-29* calls for the rationale to use its mimics in the management of AML patients [142,143]. *MiR-29b-1* interplays with AML1-ETO, the oncogenic fusion protein encoded by the chromosomal translocation t(8;21), which frequently occurs in AML; Zaidi et al. found that runt related transcription factor 1 (RUNX1, also known as AML1) and RUNX1 translocation partner 1 (RUNX1T1, also known as ETO) fusion proteins (AML1-ETO) down-regulates the expression of *miR-29b-1*, that in turn could down-regulates AML1-ETO expression by direct targeting the ETO 3′UTR. Hence, the re-introduction of *miR-29b-1* in the leukemic cells induces cellular apoptosis and inhibits cell growths [144]. High levels of *miR-181a* were associated with a better outcome in AML [145]. In vitro and in vivo experiments showed that the up-regulation of *miR-181a* reduces cells growth and increases mice survival. Those effects were obtained by the direct targeting of KRAS proto-oncogene, GTPase (KRAS), NRAS proto-oncogene, GTPase (NRAS), and MAPK1 [146]. *MiR-125b-1* is involved in the chromosomal translocation t(2;11)(p21;q23), which leads to myelodysplastic syndromes (MDS) and AML [147]; its over-expression blocks apoptosis through down-regulation of multiple genes implicated in the TP53 pathway, and gives proliferative support to human and mouse myeloid cell lines [148]. A close association between *miR-125b* with immune system has been revealed, which occurs through the regulation of cellular differentiation in germinal centers [149,150].

Several other miRNAs seems important in AML. *MiR-150* acts in vitro as tumor suppressor by activating apoptosis and inhibiting proliferation in primary AML cells, as well as in vivo by the reduction of human leukemia engraftments in nude mice through the targeting of the genes eukaryotic translation initiation factor 4B (*EIF4B*), forkhead box O4 (*FOXO4*), protein kinase C alpha *(PRKCA*), and tet methylcytosine dioxygenase 3 (*TET3*) [151]. *MiR-192* is down-regulated and its overexpression arrests cell proliferation, by negative regulating cyclin T2 (CCNT2) [152]. The expression of *miR-193b* is low in pediatric AML and constitutes a poor prognostic factor. It targets multiple factors of MAPK pathway, thus arresting cell cycle [153]. *MiR-194-5p* was found deregulated in AML patients, and its restoration induces cell differentiation and apoptosis by targeting BCL2 associated transcription factor 1 (BCLAF1) [154]. Low levels of *miR-375* are associated with a worse outcome in AML patients. Its overexpression in xenograft mice models reduces tumor size [155]. High levels of *miR-19b* [156], *miR-99a* [157] and *miR-362-5p* [158] are predictor of poor prognosis. In AML cell lines *miR-126* inhibits the apoptosis by down-regulating TNF receptor associated factor 7 (TRAF7) [159].

MicroRNAs are also involved in drug-resistance in AML. A noted example being, among which the adriamycin (ADR)-based chemotherapy resistance: *miR-125a* targets the hexokinase 2 (HK2) and the lncRNA-UCA1, which acts as a competing endogenous RNA (ceRNA). The knockdown of lncRNA-UCA1 suppresses the ADR-based chemotherapy resistance in pediatric AML patients through the restoration of the *miR-125a*/HK2 pathway [160]. Similarly, lncRNA FTX function as a sponge for *miR-342*, reducing the targeting of ALG3, alpha-1,3-mannosyltransferase (ALG3), whose aberrant level contributes to ADR-resistance development [161]. Recent reviews list all the miRNAs and their specific roles in AML [162,163,164].

### 3.3. Chronic Myeloid Leukemia (CML)

More than 95% of CML patients harbor the BCR-ABL proto-oncogene 1, non-receptor tyrosine kinase (ABL1) fusion gene (BCR-ABL1), which occurs through the t(9;22)(q34;q11) chromosomal translocation [165]. MiRNAs interplay with the signaling pathway involving the BCR-ABL1. For instance, the tumor suppressor *miR-7* directly targets the ABL1 3′UTR, and its transfection in the K562 CML cell line inhibits the proliferation, increases the apoptosis and enhances sensitivity to imatinib [166], a tyrosine kinase inhibitor (TKI) specific for the TK domain of ABL1 [167]. The BCR-ABL was found to be down-regulated also by *miR-143* [168] and *mir-320a* [169]. On the other hand, BCR-ABL1 regulates the expression of *miR-21*, precisely through the transcription factor STAT5 [170], and inhibition of this miRNA leads to decrease of proliferation, increase of apoptosis [171] and enhanced sensitivity to imatinib treatment [172]. In a BCR-ABL dose-dependent manner *miR-328* goes down during blast crisis in CML, and its restoration rescues cellular differentiation and affects the survival of leukemic blast [173]. Moreover, BCR-ABL increases also the levels of enhancer of zeste 2 polycomb repressive complex 2 subunit (EZH2) by STAT5, which in turn silences the expression of *miR-219* through DNA methylation, thus increasing its anti-apoptotic target X-linked inhibitor of apoptosis (XIAP) [174].

Other down-regulated miRNAs in CML are *miR-15a-5p*, *miR-224*, *let-7i*, and *miR-34a* [175,176,177]. Enforced expression of *miR-15a-5p* results in an inhibition of tumor cell survival and metastasis by targeting the CXCL10 [175]. The ectopic expression of the *miR-224* and *let-7i* potentiates the chemosensitivity of CML cells, by targeting the oncogene ST3 beta-galactoside alpha-2,3-sialyltransferase 4 (ST3GAL4) [176]. *MiR-34a* reduces the cell proliferation by suppressing SRC proto-oncogene, non-receptor tyrosine kinase (SRC), the activator of the zinc fingers and homeoboxes 2 (ZHX2, also known as RAF)/mitogen-activated protein kinase kinase 1 (MAP2K1, also known as MEK)/ERK signalling pathway (RAF/MEK/ERK) [177].

### 3.4. Non-Hodgkin Lymphoma (NHL)

As in other hematological malignancies, high levels of *miR-155* has been observed in the most common type of NHL, the Diffuse large B cells Lymphoma (DLBCL) [178,179]. At the molecular level, *miR-155* represses the growth-inhibitory effects of bone morphogenetic protein receptor type 2 (BMPR2) and TGF-β in DLBCL cells via direct targeting of SMAD5 [180], and, in a therapeutic view, ectopic delivery of anti-miR-155 leads to lymphoma regression in a xeno-transplant mouse model [181]. Recently it was shown that an increased expression of *miR-27b*, by the histone deacetylase 6 (HDAC6) knockdown, inhibits tumor growth in a DLBCL mouse model, by targeting the MET proto-oncogene, receptor tyrosine kinase (MET)/phosphatidylinositol-4,5-bisphosphate 3-kinase catalytic subunit beta (PIK3CB, also known as PI3K)/AKT oncogenic pathway (MET/PI3K/AKT) [182]. *MiR-26a* and *miR-181a* also display oncosuppressor features. By regulating cyclin dependent kinase 5 (CDK5) activity, *miR-26a* controls proliferation and apoptosis in DLBCL cell lines [183]. *MiR-181a* negatively modulates the NF-κB signaling and reduced the proliferation of DLBCL cells. This was more evident in cells having an activated-B-cell (ABC)-like phenotype. Concordantly, DLBCL xenografts models demonstrated a stronger effect of *miR-181a* on cell proliferation and on rate of survival in mice inoculated with ABC-like DLBCL showing an activated NF-κB signaling, rather than in those with germinal center-B cell (GCB)-like [184]. Craig, V.J. et al. [185,186] demonstrated the oncosuppressor role of *miR-34a* and its connection with the MYC proto-oncogene, bHLH transcription factor (MYC) oncogene in DLBCL. Acting as a repressor, MYC negatively regulates *miR-34a* inducing the expression of its target, FOXP1, whose deregulation contributes to DLBCL pathogenesis [185,186,187]. 

The molecular and clinical importance of the BCR signaling in DLBCL is underlined by the newly approved drugs specific for this pathway [188]. *miR-17-92* overexpression in DLBCL enhances the pathogenic BCR signaling by the direct repression of protein tyrosine phosphatase, receptor type O (PTPRO) and PPP2CA gene products, and its inhibition increases the sensitivity to cytotoxic effects of BCR-inhibitors [189]. Again, re-expression of *miR-28* dampens BCR signaling and blocks tumor growth in xenograft mice [190]. 

Follicular dendritic cells (FDCs) are critical for maintaining of germinal centers B-cell survival and their differentiation. In this context FDCs decrease the expression of BCL6 up-regulating *miR-30* family*,* while increase the expression of PRMD1 by targeting *miR-9/let-7* family [191].

Regarding Burkitt Lymphoma (BL), another subtype of NHL, the *miR-155* was interestingly found down-regulated [192]. In transgenic mice, the loss of *mir-155* has been correlated with high levels of AID protein and the frequency of the typical BL t(8;14) (q24;q32) immunoglobulin heavy locus (IGH)/MYC chromosomal translocation [193,194]. Interestingly, an inverse correlation was found among oncogene MYC mRNA expression and *miR-29* in BL cells. The expression of this miRNA was induced by DNA de-methylating agents and inhibits the protein expression of cyclin dependent kinase 6 (CDK6), DNA methyltransferase 3 beta (DNMT3B), T cell leukemia/lymphoma 1A (TCL1A, also known as TCL1) and MCL1, BCL2 family apoptosis regulator (MCL1) [195]. MYC also downregulates the expression of *miR-26a*, which in turn down-regulates EZH2, thus affecting the cell cycle progression [196].

Several miRNAs were found dysregulated in T-cell lymphoma [197,198,199,200,201], and *miR-155* is one of the most deregulated miRNA. Indeed, a phase 1 clinical trial to evaluate the MRG-106 drug, a synthetic inhibitor of *miR-155* is undergoing (NCT02580552) [202,203].

It was reported that *miR-106a-363* miRNA cluster contributes to murine T-cell lymphoma despite the activation of p27/Kip1 cell cycle inhibitor [204]. Then, *miR-150* inhibits AKT signaling and increases radiosensitivity in NK/T cell lymphoma cells [205].

### 3.5. Hodgkin Lymphoma (HL)

HL is a heterogeneous disease with several subtypes. Elevated levels of *miR-9* were found in HL cell lines [206]. Leucci et al. showed that *miR-9* contributes to the pathogenesis of HL by targeting dicer 1, ribonuclease III (DICER1) and ELAV like RNA binding protein 1 (ELAVL1, also known as HUR) and thus affecting the cytokine production; moreover, inhibition of *miR-9* in xenograft HL models leads to the reduction of tumor outgrowth [207]. *MiR-24-3p* is up-regulated in HL; it affects cell growth by targeting both cyclin dependent kinase inhibitor 1B (CDKN1B)/P27^kip1^ and MYC in HL cell lines; its inhibition increases the apoptotic cells [208]. The aberrant methylation of *miR-124a* was found to be associated with aggressive HL disease and other hematological malignancies [209,210]. Both in HL patients and in HL cell lines, the tumor suppressor *miR-34a* and *miR-203* are methylated. The re-expression of these miRNAs in HL cell lines, by using de-methylating agent, shows antiproliferative effect [211]. Moreover *miR-96, miR-182*, and *miR-183* seems to have a role in classical HL by interfering with FOXO1, an important transcription factor that regulates B cell survival and differentiation [212].

## 4. Conclusions and Future Perspectives

This review summarizes the most recent findings that correlate miRNAs to autoimmune disease and hematological malignancies outlined in Table 1. As emphasized, the co-presence of AD and HM in some patients could be the result of the de-regulation of molecular pathways and cellular processes in common between these two types of disease. Autoimmune disease could also generate an altered microenvironment that could support the development of leukemia or lymphoma cells. We described the miRNAs associated with ADs or HMs separately since, although the emerging evidence, it is still speculative considering the de-regulated miRNAs as a general and causative link between some of the ADs to their correlated HM.

Another factor that should be considered in the intricate and uncovered relationship between ADs, HMs and miRNA, is the eventual presence of viral infection of the host with an AD or HM symptoms; interactions between virus infections and the immune systems are thought to contribute to the development of several ADs [239,240,241,242] that in turn have increased HMs incidence [243,244]. Moreover, growing evidence point out to de-regulated epigenetic mechanisms as principal causes of ADs [245,246,247,248], that could be intimately related to viral infection. Between them, miRNAs can contribute to the development of the AD and cellular transformation; indeed, several de-regulated miRNAs in ADs have a critical role in several HMs. Therefore, environmental and epigenetic factors could influence immune response, ADs development and, eventually cellular transformation. For instance, the Epstein-Barr virus (EBV)-encoded nuclear antigen EBNA-2 (EBNA-2) de-regulates immune checkpoint CD274 molecule (CD274, also known as PD-L1) protein by inhibiting the *miR-34a* in B-cell lymphoma [249]. EBNA-2 also increases the expression of *miR-21*, while down-regulates *miR-146a* in B-cell lymphoma [16]. Another EBV protein, the latent membrane protein 1 (LMP1), down-regulates TCL1 by increasing *miR-29b* levels [250]. Again, the EBV encoded miRNAs are also able to regulate host immune response [251] and are associated with overall survival in CLL [252].

One of the future directions that may impact both AD and HM diagnosis and prognosis is the use of circulating miRNAs in patients of both these groups. Reconstitution of a protective miRNAs or silencing a highly expressed miRNAs could have important implications in preventing specific AD and HM, especially when the presence of an AD increased the risk of HM. An example could be *miR-155*, which high levels were observed both in AD and HM (see table); its deletion protects mice from developing collagen-induced arthritis, when immunized with type II collagen [26], and leads to lymphoma regression in xeno-transplant mouse model [181]. All this suggests that silencing of *miR-155* will benefit both AD and HM patients, in such case both RA and lymphoma patients. To silence that miRNA could be used nanoparticles coated to the antimir-155. Correct biodistribution and target specificity may prove useful in treating both AD and HM characterized by its high expression.

The increasing knowledge in the regulation of the immune response by miRNAs could find new useful therapeutic targets in the management of AD patients, and possibly diminishing their incidence of HMs.

## Figures and Tables

**Table 1:** Molecular mechanisms of microRNAs (miRNAs) in autoimmune and hematological diseases. Abbreviations: Non-Hodgkin lymphoma (NHL); programmed cell death (PDC); systemic lupus erythematous (SLE); dendritic cells (DCs); chronic myeloid leukemia (CML); systemic sclerosis (SSc); Hodgkin lymphoma (HL); rheumatic arthritis (RA); psoriasis (Ps); chronic lymphocytic leukemia (CLL); T helper 17 cells (Th17); interferon (IFN); acute myeloid leukemia (AML); idiopathic thrombocytopenic purpura (ITP), Sjögren’s syndrome (SS); lymphoproliferative syndrome (ALPS); autoimmune hemolytic anemia (AIHA); T helper 1 cells (Th1).

miRNAs	Innate Immune Cells Hyperactivation and Inflammation	Autoreactive B Cell	T Cell Imbalance	Hematologic Disease	Standardized Incidence Ratio (SIR)
*Let-7a*				Cell proliferation in NHL [191]	
*Let-7b*	pDC activation and cytokine (IFNa) production in SLE [30];				
*Let-7c*	DCs activation in SLE-like mouse models; pro-inflammatory cytokines production [41]				
*Let-7i*				Cell proliferation in CML [176]	
*miR-7*	Fibrosis in SSc [52]			Apoptosis and cell proliferation in CML [166]	SSc-CML: 1.23 [213]
*miR-9*				Cell proliferation and inflammation in HL [206,207]; cell proliferation in NHL [191]	
*miR-10a*	Pro-inflammatory cytokines production in RA [57,58]				
*miR-10b*	Chemokines receptor production in Ps keratinocytes [61]				
*miR-15a/miR-16-1 cluster*				Apoptosis and cell proliferation in CLL [109,110,111,112,113,114,115,116,117]	
*miR-15a*				Apoptosis and cell proliferation in CML [175]	
*miR-17-92 cluster*	Pro-inflammatory cytokines production in RA [59]; Chemokines production in Ps keratinocytes [60]	IgG autoantibodies production in SSc-like mouse model [79]	Th17 differentiation in SSc-like mouse model [79]	Apoptosis and cell proliferation in NHL [189]	RA-NHL: Male 2.39, Female 2.04 [214]; 1.89 [215]; 5.4 [216]; 2.34 [217]; Male 2.07, Female 1.37 [218]; 3.54 [219]; 2.27 [220]; 2.0 [23]; 3.38 [221]; 3.31 [222]; [22,223]; Ps-NHL: 2.2 [224]; 1.4 [23]. SSc-NHL: 1.18 [225]; 2.9 [226]; 2.5 [227]; 2.1 [23]; 4.14 [221]
*miR-19b*				Disease progression in AML [156]	
*miR-21*	pDCs activation and cytokine (IFNa) production in SLE [30]; Fibrosis in SSc [51]	B cells differentiation and IgG autoantibodies production in SLE [77]; IgG autoantibodies and co-stimulatory molecules production in SLE-like mouse model [78]	Pro-inflammatory cytokines production in SLE-like mouse model [78]; T cells apoptosis in Ps [86]; Th17 differentiation in RA [87]; methylation of autoimmune-associated-genes in SLE and SLE-like mouse model [88]	Disease progression and apoptosis in CLL [121,122,124,125]; apoptosis and cell proliferation in CML [170,171,172]	SLE-CLL: 1.17 [213]; SSc-CLL: nd; Ps-CLL: 1.10 [213]; RA-CLL: 1.09 [213]; SLE-CML: 1.90 [213]; SSc-CML: 1.23 [213]; Ps-CML: nd; RA-CML: 2.4 [228]; 1.43 [213]
*miR-23b*			Pro-inflammatory cytokine production in SLE or RA and SLE and RA-like mouse models [99]		
*miR-24*				Apoptosis in HL [208]	
*miR-26a*				Apoptosis and cell proliferation in NHL [183,196]	
*miR-27b*				Cell proliferation in NHL [182]	
*miR-28*				Cell proliferation in NHL [190]	
*miR-29*	Fibrosis in SSc [48,49]			Apoptosis and cell proliferation in AML [142,143,144]; apoptosis and cell proliferation in NHL [195]	SSc-AML: 1.01 [213]; SSc-NHL: 1.18 [225]; 2.9 [226]; 2.5 [227]; 2.1 [23]; 4.14 [221]; SLE-AML: nd; SLE-NHL: 7.01 [229]; 2.86 [230]; 3.50 [231]; 2.74 [232]; 15.37 [233]; 7.27 [219]; 5.0 [220]; 4.39 [234]; 5.70 [235]; 7.40 [221] 4.40 [23]; 12.10 [236]; [22,223]
*miR-30a*		B cells proliferation and IgG autoantibodies production in SLE [73]		Apoptosis in NHL [191]	SLE-NHL: 7.01 [229]; 2.86 [230]; 3.50 [231]; 2.74 [232]; 15.37 [233]; 7.27 [219]; 5.0 [220]; 4.39 [234]; 5.70 [235]; 7.40 [221] 4.40 [23]; 12.10 [236]; [22,223]
*miR-30b*	Fibrosis in SSc [50]			Apoptosis in NHL [191]	SSc-NHL: 1.18 [225]; 2.9 [226]; 2.5 [227]; 2.1 [23]; 4.14 [221]
*miR-31*	Pro-inflammatory cytokines and chemokines production in Ps [55]; keratinocyte proliferation in Ps [56]				
*miR-34a*	DCs activation in RA [39]			Cell proliferation in CML [177]; apoptosis and cell proliferation in NHL [185,186]; cell proliferation in HL [211]	RA-CML: 2.4 [228], 1.43 [213]; RA-NHL: Male 2.39, Female 2.04 [214]; 1.89 [215]; 5.4 [216]; 2.34 [217]; Male 2.07, Female 1.37 [218]; 3.54 [219]; 2.27 [220]; 2.0 [23]; 3.38 [221]; 3.31 [222]; [22,223]; RA-HL: 3.06 [215]; 4.05 [217]; 1.76 [219]; 12.82 [237]; 3.31 [222]
*miR-34b/c*				Disease progression in CLL [117]	
*miR-99a*				Disease progression in AML [157]	
*miR-106a~363Xpcl1 cluster*				Cell proliferation in NHL [204]	
*miR-124a*				Disease progression in HL [209,210]	
*miR-125a*			Treg differentiation in ITP [95]	AML [160]	ITP-AML: 3.46 [213]
*miR-125b*	Keratinocytes proliferation and differentiation in Ps [66]			Disease progression, apoptosis and cell proliferation in AML [147,148,149,150]	Ps-AML: 1.26 [213]
*miR-126*			Methylation of autoimmune-associated-genes in SLE [90]	Apoptosis in AML [159]	SLE-AML: nd
*miR-130b*	Cytokines production (regulator type I IFN pathway) in renal cells (SLE) [31]; fibrosis in SSc [33];				
*miR-138*	Proliferation in Ps [64]		Th1 differentiation in Ps [100]		
*miR-142*	pro-inflammatory cytokines production in SLE [40]	T cell activation and B cell stimulation in SLE [72]			
*miR-143*				Apoptosis and cell proliferation in CML [168]	
*miR-146a*	type I IFN pathway in SLE [36]; co-stimulatory molecules production in SS-prone mice [37];	IgG autoantibodies production in ALPS-like mouse model [76]			
*miR-146b*	chemokines receptor production in Ps keratinocyte [61]	Co-stimulatory molecules production in patients with CLL associated with AIHA [14]	Treg differentiation in ITP [94]		
*miR-148a*		Survival of immature B cells in SLE-like mouse model [74]	Methylation of autoimmune-associated-genes in SLE and SLE-like mouse model [88]		
*miR-150*	DCs activation and cytokines production in SLE-like mouse model [42]; fibrosis in lupus nephritis [43]; fibrosis in SSc [44,45]			Disease progression and cell proliferation in CLL [118]; apoptosis and cell proliferation in AML [151]; apoptosis in NHL [205]	SLE-CLL: 1.17 [213]; SSc-CLL: nd; SLE-AML: nd; SSc-AML: 1.01 [213]; SLE-NHL: 7.01 [229]; 2.86 [230]; 3.50 [231]; 2.74 [232]; 15.37 [233]; 7.27 [219]; 5.0 [220]; 4.39 [234]; 5.70 [235]; 7.40 [221] 4.40 [23]; 12.10 [236]; [22,223]; SSc-NHL: 1.18 [225]; 2.9 [226]; 2.5 [227]; 2.1 [23]; 4.14 [221]
*miR-155*	Chemokine production and pro-inflammatory chemokine receptor expression in RA monocytes [25]; pro-inflammatory cytokines production in RA synovial CD14(+) [26]; resistance to apoptosis in CD14+ RA [24]; DCs hyperactivation [27]; co-stimulatory molecules production in pDCs from SLE-like mice model [28]; fibrosis in SSc [29]; proliferation in Ps [63]	IgG autoantibodies production in SLE-like mouse model [67]; in RA [69] and in RA-like mouse model [26].	Treg cells development in SLE-like mouse model [82]; pro-inflammatory cytokines production in RA [85] and in RA-like mouse model [18]	Disease progression and cell proliferation in CLL [126,127,128,129,130,131,133]; disease progression and cell proliferation in AML [134,135,136,138,139,140,141]; cell proliferation in NHL [178,179,180,181,192,193,194]	RA-CLL: 1.09 [213]; SLE-CLL: 1.17 [213]; RA-AML: 2.4 [213,228,238]; SLE-AML: nd; RA-NHL: Male 2.39, Female 2.04 [214]; 1.89 [215]; 5.4 [216]; 2.34 [217]; Male 2.07, Female 1.37 [218]; 3.54 [219]; 2.27 [220]; 2.0 [23]; 3.38 [221]; 3.31 [222]; [22,223]; SLE-NHL: 7.01 [229]; 2.86 [230]; 3.50 [231]; 2.74 [232]; 15.37 [233]; 7.27 [219]; 5.0 [220]; 4.39 [234]; 5.70 [235]; 7.40 [221] 4.40 [23]; 12.10 [236]; [22,223]
*miR-181a*				Disease progression and cell proliferation in AML [145,146]; cell proliferation in NHL [184]	
*miR-181b*	Keratinocytes proliferation in Ps [65]			Disease progression and apoptosis in CLL [119,120]	Ps-CLL: 1.10 [213]
*miR-192*				Cell proliferation in AML [152]	
*miR-193b*				Disease progression and cell proliferation in AML [153]	
*miR-194*				Apoptosis in AML [154]	
*miR-196a*	Fibrosis in SSc [53,54]				
*miR-202*	Fibrosis in SSc [47]				
*miR-203*				Cell proliferation in HL [211]	
*miR-210*			Th1 and Th17 cell differentiation [92] and pro-inflammatory cytokines production in Ps [91]		
*miR-219*				Apoptosis in CML [174]	
*miR-224*				Cell proliferation in CML [176]	
*miR-302d*	Cytokines production (regulator type I IFN pathway) in SLE [32]				
*mir-320a*				Apoptosis and cell proliferation in CML [169]	
*miR-326*		B cells hyper activation of and IgG autoantibodies production in SLE-like mouse model [75]			
*miR-328*				Cell proliferation in CML [173]	
*miR-342*				Cell proliferation and apoptosis in AML [161]	
*miR-362*				Disease progression in AML [158]	
*miR-375*				Disease progression in AML [155]	
*miR-483*	Fibrosis in SSc [46]				
*miR-486*	Keratinocytes proliferation in Ps [62]				
*miR-574*	pDCs activation and cytokine (IFNa) production in SLE [30]				
*miR-618*	Citokines production (IFNA1) from pDCs in SSc [34]				
*miR-1246*		B cells hyper activation and co-stimulatory molecules production in SLE [71]			

## Abbreviation

SIR	Standardized incidence ratio
nd	not detected
SLE	Systemic Lupus Erythematous
RA	Rheumatic Arthritis
SSc	Systemic Sclerosis
Ps	Psoriasis
AIHA	Autoimmune Haemolytic Anemia
MS	Multiple Sclerosis
ITP	Idiopathic Thrombocytopenic Purpura
ALPS	Lymphoproliferative Syndrome
NHL	Non-Hodgkin Lymphoma
HL	Hodgkin Lymphoma
AML	Acute Myeloid Leukemia
CML	Chronic Myeloid Leukemia
CLL	Chronic Lymphoid Leukemia
DCs	Dendritic Cells
pDCs	plasmacytoid Dendritic Cells
SS	Sjögren’s syndrome
